# PMI TechNote: Bacteriophages—From the Petri Dish to Conquering the Nooks and Crannies of Difficult‐to‐Treat Infections

**DOI:** 10.1111/apm.70249

**Published:** 2026-08-25

**Authors:** Amalie Høgh Eichler, Ida Friberg Hitz, Kira Céline Koonce, Leah M. Smith, Jesper Juel Mauritzen, Nina Molin Høyland‐Kroghsbo

**Affiliations:** ^1^ Department of Immunology and Microbiology University of Copenhagen Copenhagen Denmark; ^2^ Department of Plant and Environmental Sciences University of Copenhagen Frederiksberg C Denmark; ^3^ Department of Microbiology and Immunology University of Otago Dunedin New Zealand

**Keywords:** anaerobic growth, antibiotic resistance, bacterial persistence, bacteriophage, biofilm, extracellular matrix, phage therapy

## Abstract

Bacterial infections present a challenge in clinical settings due, in part, to their remarkable diversity in physiological context and treatment resistance. Bacteriophage viruses (phages) offer renewed therapeutic promise, yet their performance in vivo often differs from expectations based on standard laboratory assays. Biofilms, with their dense extracellular matrix and spatial heterogeneity, restrict phage access, diffusion, and replication. Anaerobic niches and metabolically dormant bacterial subpopulations at the biofilm core pose additional hurdles, often rendering bacteria tolerant to both antibiotics and phages. These physiological constraints are rarely captured during phage isolation, where agar‐based plaque assays, oxygen‐rich conditions, and rapidly growing hosts inadvertently deselect phages adapted to biofilms, anaerobic zones, or dormant states. Understanding how phage–bacterial interactions vary across these niches and how antibiotics synergize with or antagonize phage activity is essential for designing therapies with improved effectiveness beyond the Petri dish. This TechNote synthesizes key challenges for phage infection dynamics in biofilms, anaerobic environments, and dormant populations, highlighting the need for isolation and screening strategies that better reflect the physiology of clinical infections.

## 1 Introduction

Antimicrobial resistance (AMR) is an urgent global health challenge, causing an estimated 1.14 million deaths annually and contributing to nearly 4.71 million deaths worldwide [1]. The World Health Organization reports that one in six laboratory‐confirmed bacterial infections in 2023 were caused by antibiotic‐resistant strains. Between 2018 and 2023, antimicrobial resistance increased in 40% of monitored pathogen‐antibiotic combinations, with the most concerning trends observed among Gram‐negative pathogens [2]. To prevent the devastating impact of infectious disease in the post‐antibiotic era, there is an urgent need for innovations tailored to infection physiology.

As AMR rises, the use of bacteriophage viruses (phages) to selectively kill bacteria, known as phage therapy, is reemerging as a promising alternative or complement to antibiotics. Phage therapy is generally considered safe for difficult‐to‐treat infections [3, 4]. Phages infect their hosts with high specificity, often targeting only a subset of strains within a given species. Phages selectively attach to bacterial surface structures, such as lipopolysaccharide, outer membrane proteins, pili, or flagella, and subsequently deliver their genomic material into the cell [5]. Next, the phage typically replicates either through a lytic cycle, which culminates in host cell destruction and release of phage progeny, or through a lysogenic cycle, in which the phage genome integrates into the host chromosome as a prophage and persists without immediately killing the cell [3].

Across 100 consecutive personalized phage therapy cases, clinicians observed improvement in 77% of patients and successful eradication of the target bacterium in 61%, highlighting the promise of tailored phage approaches for otherwise difficult‐to‐treat infections [6]. While personalized phage therapy shows encouraging clinical outcomes, many of the infections most in need of such interventions are biofilm‐associated, which are notoriously difficult to treat, in part due to increased antibiotic tolerance. Biofilms are microbial communities embedded in a semi‐structured matrix that exists as part of a dynamic host‐pathogen partnership, shaped by microbial interactions, host responses, and environmental factors [7]. In short, the biofilm matrix consists of bacteria, often from multiple species, surrounded by self‐produced extracellular polysaccharides, membrane vesicles, DNA, and proteins, including adhesins, structural proteins, and amyloid fibers that provide a strong scaffold [8]. Some biofilms also gain structural strength from long and negatively charged filamentous phages [9]. Biofilms fundamentally reshape bacterial physiology and form dense barriers that alter, for example, how phages access and infect bacteria. Yet most therapeutic phages are not selected for their ability to perform within biofilms.

**FIGURE 1:**
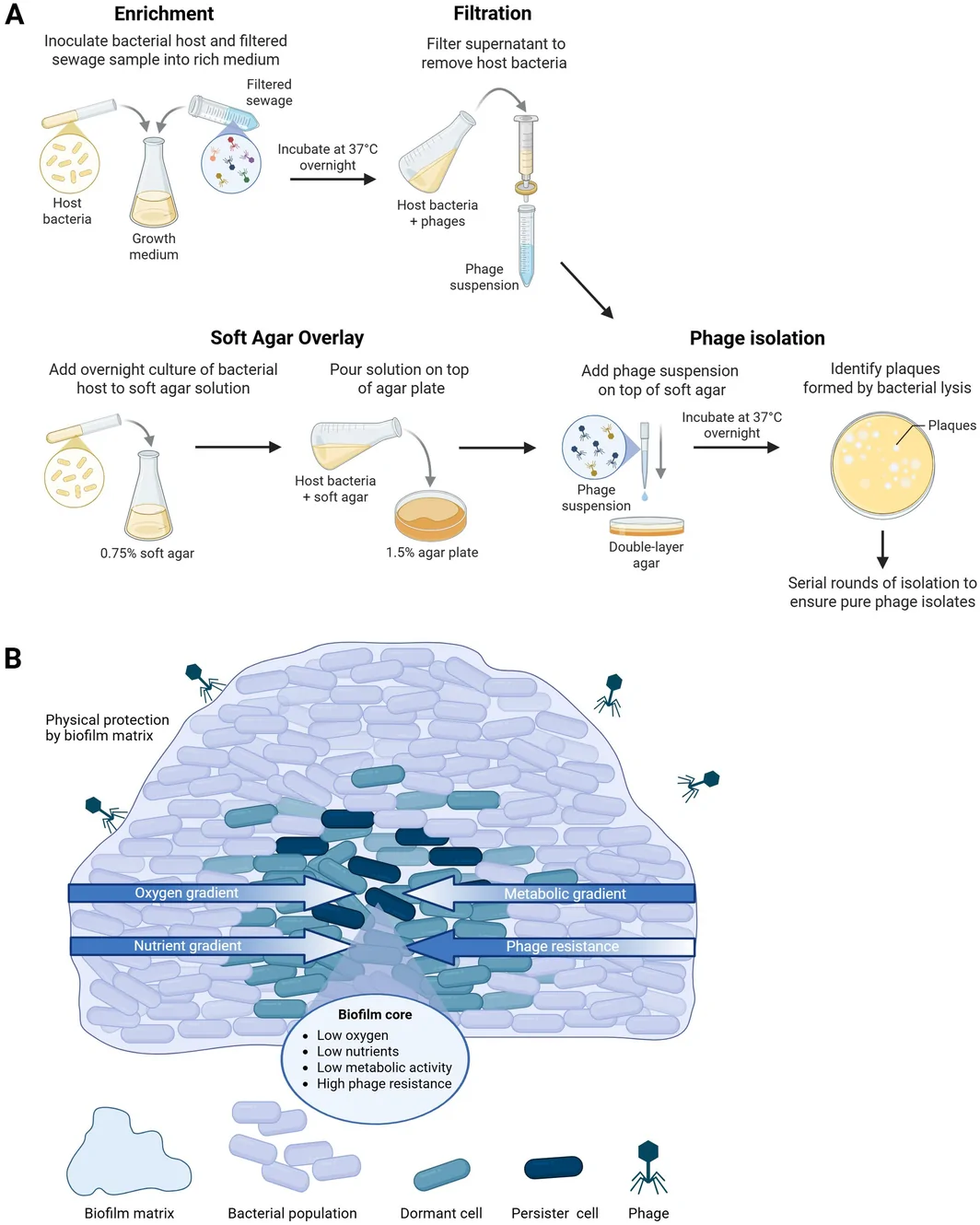
*Standard phage isolation and key biofilm‐related physiological challenges for phages*. (A) Standard phage isolation procedure. A phage‐rich sample (e.g., filtered sewage) is mixed with a desired bacterial host strain in liquid growth media. After incubation, the supernatant is filtered to remove bacteria. The phage suspension is spread on a double agar overlay with the host strain and incubated. The lysis zones (plaques) indicate bacterial killing formed by individual infective phages that can be further isolated. (B) The biofilm challenge. Due to physical extracellular barriers and altered bacterial physiology, biofilms pose unique challenges to phage access, diffusion, and infection dynamics. Bacteria near the biofilm surface have greater access to oxygen and nutrients and therefore generally remain more metabolically active, whereas those deeper in the biofilm core generally exhibit reduced metabolic activity and may enter various stages of dormancy or persistence, lowering their susceptibility to phage infection or killing. Created with BioRender.

Despite the importance of biofilm‐associated infections, phage isolation and characterization is typically performed under aerobic, nutrient‐rich, planktonic conditions, creating strong selection biases for phages that replicate rapidly in laboratory environments rather than in clinically relevant niches such as biofilms, anaerobic zones, or dormant states. Standard phage isolation workflows begin with phage‐rich samples (e.g., filtered sewage) mixed with a *single* bacterial strain in well‐aerated liquid media to enrich rapidly proliferating phages specific to the host bacterium. After incubation, the supernatant is filtered and spotted in a plaque assay, via a double agar overlay method, that visualizes zones of bacterial lysis formed by individual infective phages on Petri‐plates (Figure 1A). Phages that are not strictly lytic and those carrying undesirable traits, such as toxin or antibiotic resistance genes, are excluded based on genomic screening, before moving to further in vitro and in vivo testing. While efficient, there is a tradeoff. These steps typically overlook clinically relevant biophysical barriers, oxygen gradients, and altered bacterial physiology in biofilms, likely contributing to phage panels that initially perform well on plates but less so in difficult‐to‐treat infections [10, 11].

Here, we outline key barriers that phages face in complex biofilm infection niches (Figure 1B). We focus on biofilms, anaerobic environments, and metabolically dormant bacterial subpopulations to highlight some of the factors that constrain therapeutic success beyond the Petri dish.

## 2 Biofilm Barriers to Phage Infection

By forming dense extracellular barriers and altering bacterial physiology, biofilms impose unique constraints on phage access, diffusion, and infection dynamics while simultaneously shielding the bacteria from the host's immune defense and antibiotics [8, 9, 12, 13, 14]. In addition to biophysical barriers, specific matrix components can further enhance protection. For instance, the biofilm amyloid fiber network protects *Escherichia coli* cells by not only blocking phage penetration into the biofilm but also by sequestering phage particles, preventing attachment to the bacterial cell surface [15]. Still, the same matrix features that hinder some phages can also be co‐opted or bypassed by others, highlighting a spectrum of phage strategies for engaging with biofilm‐embedded hosts. The *Pseudomonas aeruginosa* phage Clew‐1 preferentially infects biofilm‐dwelling cells and relies on the Psl exopolysaccharide biofilm component for infection [16]. Additionally, some phages have evolved to overcome the biofilm barrier by producing biofilm‐degrading enzymes such as depolymerases [17], allowing phages to dig into the biofilm and thereby successfully infect their host [18].

Beyond protection by the biofilm matrix, the altered physiological conditions within the biofilm also shape phage‐bacterial interactions. For instance, when *P. aeruginosa* biofilms mature, the sessile bacteria no longer produce flagella, which are required for motility [19], so this likely renders those bacteria resistant to a range of flagellotropic phages.

These biofilm‐driven shifts in bacterial access and physiology are only one layer of complexity; deeper within the biofilm, steep oxygen gradients carve out anaerobic niches where phage‐bacterial dynamics operate under entirely different rules.

## 3 Phage–Bacterial Dynamics in Anaerobic Niches

Oxygen gradients within biofilms create metabolically stratified niches that fundamentally alter bacterial physiology and, consequently, shape the outcomes of phage‐bacterial interactions. Bacteria near the biofilm surface can access oxygen and nutrients more readily and therefore remain metabolically active, whereas the biofilm core becomes oxygen depleted. Here, the bacteria exhibit reduced metabolic activity and some, including *P. aeruginosa* , depend on denitrification or arginine fermentation for anaerobic respiration [20, 21].

The reduced metabolic activity makes antibiotics largely ineffective, as most antibiotics rely on metabolically active bacteria to exert their effects [22]. One study used the phage PAK_P1 to examine how phage infection of *P. aeruginosa* differs under anaerobic conditions. Importantly, phage adsorption decreased by 66% in the anaerobic condition, suggesting that low‐oxygen environments may alter phage receptor expression. Despite the reduced adsorption, neither the PAK_P1 latent period nor its lytic efficiency was affected [23].

In *E. coli* , phage T1 failed to replicate under anaerobic conditions, whereas T2 or T4 remained unaffected. However, supplementing anaerobic cultures with glucose, pyruvate, and oxalacetate restored T1 replication. These findings indicate that phage replication in *E. coli* depends strongly on host metabolic activity [24]. Similarly, Villamizar et al. showed that oxygen availability strongly shapes phage infection dynamics. Under anaerobic conditions, phage ϕSan23 displayed a markedly reduced burst size and an extended lytic cycle, producing roughly half the amount of progeny per infected *Salmonella* cell. One contributing factor may be altered activity of the bacterial replication machinery: during anaerobiosis, host replication may slow or stall, reducing the rate at which phages can replicate and consequently delaying host killing. Interestingly, despite this slower replication, the emergence of phage‐resistant mutants was lower in anaerobic cultures [25]. Overall, anaerobic conditions modulate phage replication and host resistance in phage‐ and nutrient‐dependent ways, emphasizing the importance of accounting for oxygen availability and bacterial physiology at infection sites where phage therapy is applied.

**FIGURE 2:**
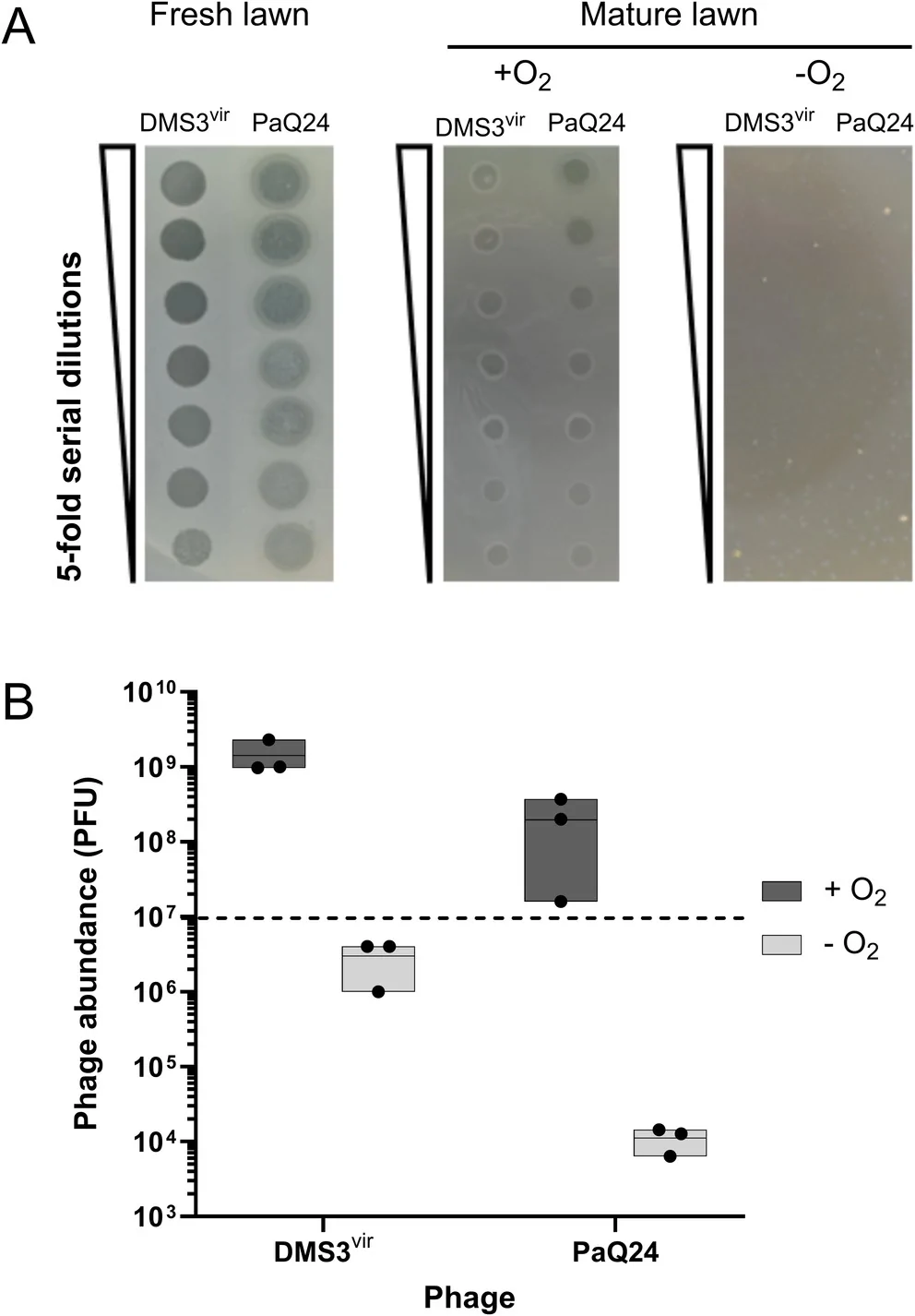
*PaQ24 is highly dependent on oxygen for replication*. (A) Plaque assays of phages DMS3^vir^ and PaQ24 on *P. aeruginosa* PA14: Fresh standard lawn (left), mature lawn grown with oxygen (center), or anaerobically (right). (B) Phage abundance within representative plaques presented as PFU quantified from clearing zones formed by DMS3^vir^ and PaQ24 on mature lawns. The dashed line indicates the phage abundance used as input. Standard deviation, *n* = 3 replicates.

As mentioned earlier, routine phage isolation procedures select for phages that kill a desired bacterium with high efficiency under aerobic nutrient rich conditions, introducing the risk of selecting for phages with reduced killing activity when their host is growing anaerobically [10, 11]. Indeed, we subjected our *P. aeruginosa* phage panel isolated as in Figure 1A, to plaque assays on mature lawns of *P. aeruginosa* UCBPP‐PA14. We noted one newly isolated phage that performed particularly poorly under anaerobic conditions. Phage DMS3^vir^ [26] and our phage PaQ24 formed distinct lysis zones on both fresh and mature bacterial lawns (Figure 2A). Both phages replicated on the mature lawns, yielding 150‐fold and 20‐fold higher phage counts, respectively, compared to the total phage input at 10^7^ plaque forming units (PFU). In contrast, on anaerobic mature lawns, phage‐mediated lysis was diminished, and the DMS3^vir^ and PaQ24 plaques contained 3‐fold and 900‐fold lower PFU, compared to the total phage input, indicating substantially impaired replication, particularly for PaQ24 (Figure 2B). These results underscore that while phages may perform robustly in nutrient‐rich, oxygenated environments, their apparent efficacy can diminish substantially in anaerobic biofilm‐like settings, a phenomenon that becomes even more pronounced when bacteria enter dormant persister states.

## 4 Antibiotic Persistent and Dormant Bacteria—A Unique Physiological Challenge

Persister cells pose a major barrier to achieving complete infection clearance. Persister cells are a subset of a bacterial population that enter a transient non‐growing or slow‐growing state, allowing them to tolerate antibiotics without acquiring genetic resistance. Unlike antibiotic resistance, persistence is reversible, and once a persister cell resumes growth, it regains antibiotic susceptibility. Antibiotic persistence is often linked to dormancy, a state of reduced metabolism and growth that increases antibiotic tolerance, but persistence also arises via other mechanisms, including toxin‐antitoxin imbalance [27].

Phage infection can also trigger bacterial persistence or dormancy [28], creating a potentially problematic scenario in which phage therapy inadvertently induces transient antibiotic resistance. One mechanistic example involves the adaptive anti‐phage defense system CRISPR‐Cas, which can be induced upon phage infection [29]. Notably, CRISPR‐Cas13 activity induces dormancy in infected cells, preventing successful phage replication [30]. This replication failure likely stems from the slowed or stalled growth associated with persistence, giving the cells additional time to expand their CRISPR arrays and eliminate the phage before it can complete its life cycle [31, 32]. Consistent with this, we recently found that *E. coli* persister cells acquire CRISPR immunity 10‐fold more frequently than the general bacterial population [33]. Thus, persister cells, in addition to being notoriously antibiotic‐tolerant, may be in a state of heightened anti‐phage immune capacity, promoting the survival of the population and potentially limiting the efficacy of phage therapy.

Our understanding of how dormant cells respond to phage infection remains limited. The phages λ and T4 readily infect dormant, stationary‐phase *E. coli* , where early stages of the infection cycle proceed, but phage replication is halted until the host resumes normal growth upon nutrient addition. If the host's intrinsic defense systems fail to eliminate the phage, replication resumes once the bacterium returns to active growth following nutrient replenishment [34, 35]. This finding aligns with in vitro experiments showing that progeny production of λ, P1, and T4 phages can be completely blocked by starving infected cultures. Replenishing glucose to these starved cells reinitiates phage development, indicating that carbon depletion stalls phage replication [36].

However, dormancy does not universally halt phage development; certain phages have adapted to bypass these metabolic constraints and replicate directly in inactive cells. *E. coli* phage T7 selectively inhibits the stationary phase RNA polymerase E*σ* ^S^, enabling productive infection even in non‐growing cells [37]. The *Staphylococcus epidermidis* phage SEP1 similarly replicates in stationary‐phase cells by activating transcription of host genes involved in metabolism and translation—cellular functions essential for phage propagation [38]. Likewise, the phage Paride was isolated directly on dormant *P. aeruginosa* cultures, and it can lyse metabolically dormant, antibiotic‐resistant cells [39]. These findings demonstrate that isolating phages capable of eliminating dormant bacteria is feasible, given the right isolation parameters.

## 5 Phage–Antibiotic Synergy and Antagonism

Phage therapy is generally administered with antibiotics, and their combined effects can span from strong synergy to clear antagonism, shaped by the particular phage, antibiotic mode of action, and physiological state of the host. Maffei et al. showed a synergistic effect of combination treatment with phage Paride and meropenem. The treatment eradicated deep‐dormant *P. aeruginosa* cultures in vitro and significantly reduced resilient tissue cage implant infections in mice, underlining the promising potential of this phage‐antibiotic combination treatment [39]. Phage T4 combined with cefotaxime is significantly more effective at eradicating *E. coli* biofilms compared to the antibiotic alone [40]. A combination of T4 with tobramycin resulted in a 99% reduction in antibiotic‐resistant cells and a 39% reduction of phage‐resistant cells in *E. coli* biofilms. However, in *P. aeruginosa* biofilms, combining tobramycin with phage PB1 offered no improvement over tobramycin alone [41], whereas phages NP1 and NP3 synergize with ceftazidime and ciprofloxacin against *P. aeruginosa* biofilms, but not with colistin or gentamicin, suggesting that synergy emerges only when the mode of action of the antibiotic aligns with the phage's infection strategy [42]. Phages likely perform better in combination with bactericidal antibiotics, which actively kill bacteria, rather than bacteriostatic ones. This is because bacteriostatic antibiotics generally reduce bacterial growth and biosynthetic activity, thereby limiting phage replication. Additionally, the specific phage receptor can be essential for such synergy. One example is *P. aeruginosa* phage OMKO1, which uses the outer membrane porin M of the multidrug efflux systems MexAB and MexXY as its receptor. OMKO1 attack selects for efflux pump mutants, which consequently become more susceptible to multiple classes of antibiotics [43].

However, specific combinations of phages and antibiotics can exhibit antagonism, leading to reduced bacterial clearance. Phage infection can induce bacterial stress, resulting in increased antibiotic resistance [44] and virulence activation [45]. The timing and order of administering phages and antibiotics also appear to play a central role. Treating *S. aureus* biofilms with lytic phage SATA8505, antibiotics, or both simultaneously reduces biofilm associated cells only minimally. In contrast, applying the phage before the antibiotic effectively reduced biofilm formation [44]. However, in current clinical practices, patients would most likely receive antibiotics prior to and during phage therapy, as phages are used in some countries as compassionate‐use treatment for patients with limited therapeutic options [6].

Taken together, phage‐antibiotic synergy generally outweighs antagonism, a conclusion reinforced by Pirnay et al., reporting that in 100 consecutive phage therapy cases, treatment success was far higher when phages were combined with antibiotics, with eradication being 70% less likely in the absence of antibiotics [6].

## 6 Discussion

Phage therapy holds substantial promise for treating infections, even those that evade conventional antibiotics. Here, we highlight the importance of the physiological context of the infection niche for shaping outcomes of phage‐bacterial interactions. Biofilms, anaerobic niches, and dormant or persister states each impose distinct constraints on phage diffusion, adsorption, and replication, meaning that phages isolated under standard aerobic, nutrient rich conditions may not perform optimally in clinically relevant environments. While some phages can penetrate or exploit biofilm structures, replicate under oxygen limitation, or even replicate in dormant cells, many others are stalled by these conditions, underscoring the need for isolation and screening strategies that reflect infection physiology more closely. Moreover, because phage‐host interactions are highly strain‐specific and strongly influenced by environmental conditions, understanding the biological traits that drive these diverse outcomes is essential for selection of phages that perform optimally under the desired conditions of each infection niche.

Across these challenging scenarios, combination therapy emerges as a particularly powerful strategy. Although antagonism can occur, especially when antibiotics suppress phage propagation or when phage infection induces bacterial stress responses, the overall pattern across experimental and clinical studies points toward synergy. Mechanistic alignment between phage infection strategy and antibiotic mode of action, as well as treatment order, can dramatically enhance bacterial clearance. Clinical data underlines this trend: in a cohort of 100 consecutive phage therapy cases, infection eradication was less likely when phages were administered without antibiotics [6]. These observations emphasize that successful phage therapy depends not only on selecting the right phages but also on understanding the metabolic and spatial landscape of the infection and designing combination regimens that enhance, rather than hinder, phage activity.

Together, these insights argue for a shift toward phage discovery, characterization, and therapeutic design that explicitly incorporates the environmental and physiological complexity of real infections.

Phage “cocktails” that combine multiple phages using complementary receptors are often applied in therapeutic settings to overcome the problems of limited phage host range and to reduce the emergence of phage resistance [46]. Similarly, strategically designing phage cocktails with complementary functional traits to circumvent biofilms, anaerobic niches, and dormant populations may be used to target difficult‐to‐treat infections. Inclusion of phages encoding specific depolymerases can facilitate biofilm matrix degradation and enhance penetration of antibiotics and other phages [17]. Moreover, including both phages that effectively target metabolically active cells at the biofilm periphery and phages capable of killing dormant or persister cells in deeper, oxygen‐ and nutrient‐limited regions helps ensure efficacy in these environments. Although dormant and persister cells remain intrinsically challenging targets, phage cocktails may indirectly address these populations through lysis of active cells at the biofilm surface and disruption of the biofilm structure. This process in turn releases nutrients and thereby promotes reactivation of dormant cells, rendering them more susceptible to antibiotics and phage infection. In other words, it may be beneficial to design phage cocktails to account for the complexity of in vivo infection environments, highlighting the need for isolation and screening strategies that extend beyond conventional laboratory conditions (Figure 1A).

Standard phage isolation workflows may inadvertently select against phages that thrive in clinically relevant niches. Phages that depend on biofilm components for infection are missed because these structures are largely absent in planktonic, nutrient rich cultures [16]. For example, flagellotropic phages, which may be uniquely suited for treating urinary tract infections, are deselected in standard plaque assays where high agar content suppresses flagella production. Also, biofilmtropic phages, such as *P. aeruginosa* phage Clew‐1, which perform poorly on standard plates yet exhibit potent activity in vivo, are generally overlooked [16].

One way to counter this bias is phage training, in which phages are repeatedly passaged under desired conditions, such as biofilms [47, 48], allowing directed evolution to enhance traits like biofilm recognition, host range, or killing efficiency. Ultimately, because phage therapies are deployed in vivo, incorporating physiologically relevant parameters early in the phage isolation and screening pipeline will increase the chance that phage candidates selected in vitro retain efficacy in the complex environments of difficult‐to‐treat infections.

Co‐administering antibiotics with biofilm‐degrading phages or phages that can reactivate metabolism of dormant bacteria can in some cases enhance antibiotic penetration and sensitize otherwise protected or dormant bacteria, as shown for the *S. epidermidis* phage SEP1 [38]. Such synergy may enable lower antibiotic doses or resensitize the bacteria to antibiotics while improving killing efficiency and limiting resistance selection. Moreover, when phage resistance does arise, the resulting mutants are often more susceptible to antibiotics, our immune system, or less virulent, easing clearance by both antibiotics and the immune system [43].

## 7 Gaps in Knowledge and Outlook

Despite rapid progress, major gaps still limit the optimization of phage therapy for complex infections. How biofilm structure, oxygen gradients, and spatial heterogeneity shape phage diffusion, adsorption, and replication in vivo remains poorly understood, as most studies rely on streamlined in vitro systems. Likewise, the susceptibility of dormant and persister cells to different phages, and how phage infection itself influences persistence, phage defenses, and other stress responses, remains largely unresolved. The mechanistic basis of phage–antibiotic synergy versus antagonism is also incompletely defined, particularly how treatment order, antibiotic class, and bacterial physiological state interact across infection niches. Standard phage isolation pipelines introduce strong selection biases, yet notable efforts for discovering or evolving phages adapted to biofilms, anaerobic environments, and dormant populations show high promise. Finally, clinical data remain limited, but recent studies systematically analyzing outcomes of large numbers of phage therapy cases offer unique opportunities of exploring how in vivo phage resistance, immune interactions, and patient‐specific physiology shape treatment outcomes, and how to design combination regimens that remain robust across diverse infection contexts.

## 8 Concluding Remarks

Advancing phage therapy is urgent. Meeting the biological hurdle of this challenge benefits from experimental systems that better capture the physiological complexity of difficult‐to‐treat infections. Integrating biofilm structure, oxygen limitation, and metabolic heterogeneity into phage isolation and training pipelines will enable selection of phages that remain effective beyond planktonic, aerobic conditions. At the same time, a deeper mechanistic understanding of how phages interact with persistence pathways, phage defense systems, and different antibiotic classes, combined with coordinated clinical and laboratory studies that clarify how in vivo phage resistance, immune responses, and infection‐specific factors shape phage therapy outcomes, will help guide the design of reliably synergistic and broadly robust phage–antibiotic therapies. Together, these efforts position the field to overcome some of the physiological hurdles required to further enhance phage therapy outcomes.

## 9 Methods

*P. aeruginosa* UCBPP‐PA14 (PA14) was grown overnight at 37°C at 300 rpm in lysogeny broth (LB) (10 g l^−1^ tryptone, 5 g l^−1^ yeast extract, and 10 g l^−1^ NaCl). Phage PaQ24 was isolated from a 0.2 μM filtered sample from Lynetten wastewater treatment plant, using PA14 as a host, as in Figure 1. For plaque assays, the PA14 lawn was prepared by mixing 50 μL of overnight culture with 5 mL of molten soft agar (0.75% agar) and poured onto LB agar plate. Under anaerobic conditions, the soft agar and LB agar were supplemented with 100 mM KNO_3_ and plates were incubated in Oxoid AnaeroJar 2.5 L (ThermoFisher Scientific). The plates were incubated for 24 h at 37°C with or without oxygen, to form mature lawns. 5‐fold serial dilutions of DMS3^vir^ [26] and PaQ24 were prepared in SM buffer (100 mM NaCl, 8 mM MgSO_4_, 50 mM Tris HCl pH 7.5, and 0.01% gelatin), and spotted onto the lawns and incubated for 24 h at 37°C with or without oxygen. The clearing zones were carved out using the base of a pipette tip, mixed with SM buffer, filter‐sterilized, 5‐fold serially diluted and applied in a standard plaque assay to quantify PFU/mL. GraphPad Prism (v. 10.4.2) was used to generate graphs and run statistical analyses. GraphPad Software, Boston, Massachusetts USA, www.graphpad.com. LLM was used for language editing of the manuscript.

Figure 1 by Amalie Høgh Eichler and Kira Céline Koonce was created in BioRender https://biorender.com, licensed under CC BY 4.0.

## Funding

This work was supported by Danmarks Frie Forskningsfond, 1054‐00099B; Novo Nordisk Fonden, NNF24OC0089658; Marsden Fund, MFP‐23‐UOO‐095; Royal Society Te Apārangi and L’Oréal‐UNESCO for Women in Science.

## Ethics Statement

The authors have nothing to report.

## Conflicts of Interest

The authors declare no conflicts of interest.

## Data Availability

The Phage PaQ24 genome is deposited at NCBI (submission ID #3049737). The data that support the findings in Figure 2 are available from the corresponding authors upon reasonable request.
